# Differential expression of miRNAs in skeletal muscles of Indian sheep with diverse carcass and muscle traits

**DOI:** 10.1038/s41598-020-73071-7

**Published:** 2020-10-01

**Authors:** Mandeep Kaur, Ashish Kumar, Naveen Kumar Siddaraju, Mohamed Nadeem Fairoze, Pooja Chhabra, Sonika Ahlawat, Ramesh Kumar Vijh, Anita Yadav, Reena Arora

**Affiliations:** 1 ICAR-National Bureau of Animal Genetic Resources, Karnal, Haryana 132001 India; 2 Karnataka Veterinary Animal and Fisheries Sciences University, Bangalore, 560024 India; 3 Kurukshetra University, Kurukshetra, Haryana 136119 India

**Keywords:** Animal biotechnology, Functional genomics

## Abstract

The study presents the miRNA profiles of two Indian sheep populations with divergent carcass and muscle traits. The RNA sequencing of *longissimus thoracis* muscles from the two populations revealed a total of 400 known miRNAs. Myomirs or miRNAs specific to skeletal muscles identified in our data included oar-miR-1, oar-miR-133b, oar-miR-206 and oar-miR-486. Comparison of the two populations led to identification of 100 differentially expressed miRNAs (p < 0.05). A total of 45 miRNAs exhibited a log_2_ fold change of ≥ ( ±) 3.0. Gene Ontology analysis revealed cell proliferation, epithelial to mesenchymal transition, apoptosis, immune response and cell differentiation as the most significant functions of the differentially expressed miRNAs. The differential expression of some miRNAs was validated by qRT-PCR analysis. Enriched pathways included metabolism of proteins and lipids, PI3K-Akt, EGFR and cellular response to stress. The microRNA-gene interaction network revealed miR-21, miR-155, miR-143, miR-221 and miR-23a as the nodal miRNAs, with multiple targets. MicroRNA-21 formed the focal point of the network with 42 interactions. The hub miRNAs identified in our study form putative regulatory candidates for future research on meat quality traits in Indian sheep. Our results provide insight into the biological pathways and regulatory molecules implicated in muscling traits of sheep.

## Introduction

MicroRNAs (miRNAs) are the most recently discovered regulatory molecules that hold promise to be used as biomarkers. These are small (17–22 nucleotides) non-coding RNAs that are highly conserved across species^1^. A single miRNA is capable of targeting several genes, so the study of miRNAs provides an enhanced perspective of gene regulatory mechanisms, than that obtained from mRNAs or single genes. The role of miRNAs has been implicated in disease, growth and metabolism of skeletal muscle^2^. Their role in muscle cell proliferation and development has been well established^3,4^. Several reports are available describing the miRNA expression in skeletal muscles of cattle^5,6^, pig^7^, goat^8,9^ and sheep^10,11^. Such studies have contributed to a better understanding of the regulatory role of miRNAs in muscling traits. The hyper-muscling in Texel sheep is a result of altered sequence of target site for miR-1 and miR-206, which causes inhibition of the myostatin gene^12^. Polymorphisms have been identified in muscle specific miRNAs (myomirs) that are associated with muscle and meat quality traits in pigs^13^. MicroRNAs have also been associated with intramuscular adipocyte differentiation in cattle, buffalo, sheep and poultry^14–17^. The importance of miRNAs in regulating meat tenderness and intra-muscular fat in beef has been well elucidated^18–21^.

Since muscles form meat, it would be beneficial to know the molecular drivers that regulate their development and metabolism. Several meat type sheep breeds are found in India. Well known among them are Madgyal, Deccani, Bandur (Mandya), Hassan and Kilakarsal^22^. Bandur sheep are distributed in Karnataka and are known for their favourable organoleptic meat quality^23^. However, another sheep population (local sheep), found in the same area, are not preferred by consumers, even though the fodder, management and geographical conditions are same for both. Earlier investigations have established that the skeletal muscles of Bandur sheep have low shear force values^23^. Significant differences in back fat thickness, colour and muscle tenderness have also been reported between Bandur and local sheep^24^. Although the mRNA of skeletal muscles of Bandur and local sheep have been profiled^24^, no information is available on the differential expression of miRNAs regulating their expression. Therefore, the study was taken up with the aim to elucidate the regulatory miRNAs in Bandur and local sheep populations with diverse carcass and muscle traits.

## Results

### Summary of miRNA sequencing data

Each library from the skeletal muscles of Bandur and local sheep generated 18 to 30 million raw reads with 46–48% GC content (Table 1). The raw sequence data have been submitted to the NCBI Sequence Read Archive with Accessions SRR6346733–SRR6346740. Since only 106 precursors and 153 mature miRNAs were available in miRBase at the time of submission of this manuscript, the reads were also mapped to Human (GRCH38), as well as Bovine (ARS-UCD 1.2) reference assemblies. Mapping rate was 84% and 78% for human and bovine, respectively. A total of 400 known miRNAs were identified across all samples, of which 67, 320 and 13 were from the human, bovine and ovine database respectively. In Bandur sheep samples, 499 novel miRNA could be identified. More than 30% of the miRNAs in Bandur and local sheep had an expression of ≥ 1000 RPKM. Myomirs or miRNAs specific to skeletal muscles identified in our data included oar-miR-1, oar-miR-133b, oar-miR-206 and oar-miR-486.

**Table 1:** MicroRNA data statistics of skeletal muscles of Local and Bandur sheep.

Sample no	Raw reads	GC Content	Mapping% *Homo sapiens* (GRCH38	Mapping% *Bos taurus* (ARS-UCD 1.2)	Mapping % *Ovis aries* (oar_v3.1)
Local 1	24,000,000	47	74.47	77.91	75.70
Local 2	18,000,000	47	68.81	73.02	70.32
Local 3	20,202,020	48	76.53	79.71	74.61
Local 4	20,106,060	48	79.48	82.24	80.47
Bandur 5	30,000,000	46	92.45	75.65	74.61
Bandur 6	19,350,000	46	93.85	80.20	79.35
Bandur 7	20,750,000	46	93.79	80.45	79.63
Bandur 8	19,503,030	46	93.15	78.46	77.55

### Differentially expressed miRNAs

Of the 400 known miRNAs in our study, 100 were found to be significantly differentially expressed (p_adj_ ≤ 0.05). Out of these, 49 were up-regulated and 51 were down-regulated in Bandur sheep. Some of the up-regulated miRNAs included oar-miR-185, oar-miR-107, oar-let-7d, oar-let-7b and oar-let-7e while oar-miR-10b, oar-miR-143, oar-miR-30, oar-miR-10a and oar-miR-23a were down-regulated in Bandur. The four myomirs detected in our data were found to be differentially expressed. Myomirs, oar-miR-1 and oar-miR-206 were over-expressed while oar-miR-133b and oar-miR-486 showed lower expression in Bandur sheep. Table 2 lists the miRNAs differentially expressed in Bandur that are known to be associated with muscle growth and development in sheep^11^.

**Table 2:** Differentially expressed miRNAs (p_adj_ ≤ 0.05) in Bandur related to sheep muscle growth and development.

ID	Fold change	log_2_ fold change
mir-125a	5.5093	2.4619
mir-125b-1	0.0830	− 3.5915
mir-133b	0.1590	− 2.6528
mir-199b	0.5338	− 0.9056
mir-1–1	1.7811	0.8328
mir-1–2	1.8364	0.8769
mir-21	16.0438	4.0039
mir-221	11.1953	3.4848
mir-222	0.4498	− 1.1528
mir-23a	0.0138	− 6.1751
mir-23b	0.7339	− 0.4463
mir-27b	0.0303	− 5.0430
mir-34a	2.4465	1.2907
mir-494	14.4596	3.8540
mir-206	83.9969	6.3923
mir-486	0.0230	− 5.4442

### Prediction of target genes and pathways

**Figure 1:**
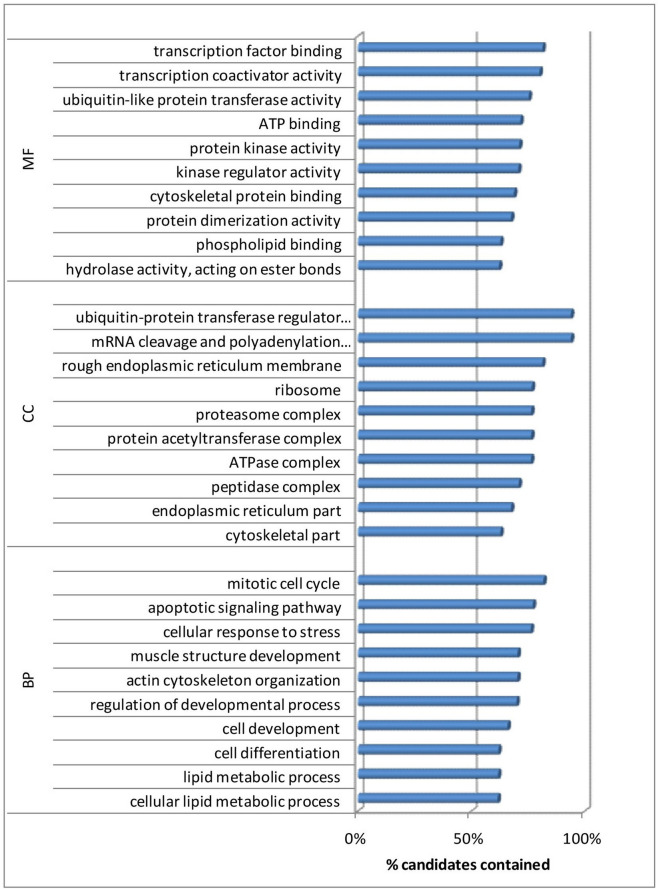
Top 10 gene ontology terms for biological process (BP), cellular component (CC) and molecular function (MF) associated with muscle traits in Indian sheep.

A total of 11,062 target genes were identified for both up and down regulated miRNAs. The list of genes was used as input for over-representation analysis using ConsensusPathDB^25,26^. More emphasis was laid on GO terms associated with muscle biology and /meat traits. The most relevant GO terms for biological process included transcription factor binding, ubiquitin-like protein transferase activity, protein kinase activity, phospholipid binding etc. The enriched cellular components were cytoskeletal part, endoplasmic reticulum part, peptidase complex, protein acetyl transferase complex, proteasome complex, ribosome, ubiquitin-protein transferase regulator activity etc. Significant molecular functions associated with muscle traits were hydrolase activity, protein dimerization activity, cytoskeletal protein binding, kinase regulator activity, ubiquitin-like protein transferase activity, transcription factor binding etc. (Fig. 1).

The predicted target genes were used to identify biological pathways using pathway-based sets (p ≤ 0.01) incorporated in ConsensusPathDB^25,26^. The top 25 canonical pathways are listed in Table S1. The differentially expressed target genes were associated with metabolism of proteins, lipids, RNA, post translational protein modification, EGFR1, Signaling by Rho GTPases, Signaling by Receptor Tyrosine Kinases, miR-targeted genes in muscle cell, PI3K-Akt signaling pathway, Cellular responses to stress etc.

All the up-regulated miRNAs in Bandur sheep were used for gene target prediction and genes associated with tenderness/meat quality (identified from published literature) were filtered from the dataset. The genes list was then compared with our previous data on mRNA of Bandur sheep^24^ and only those genes that were differentially expressed were selected. A total of 32 miRNAs were identified as potential candidates for regulating meat tenderness (Table S2). On comparing the target genes for these miRNAs with previous data on mRNA^24^, it was observed that *HSPA8* was a common target gene for 9 miRNAs while *CASP3* and *CAPN15* were targets of 8 miRNAs each.

### miRNAs with log2 fold change ≥ ( ±) 3.0

**Figure 2:**
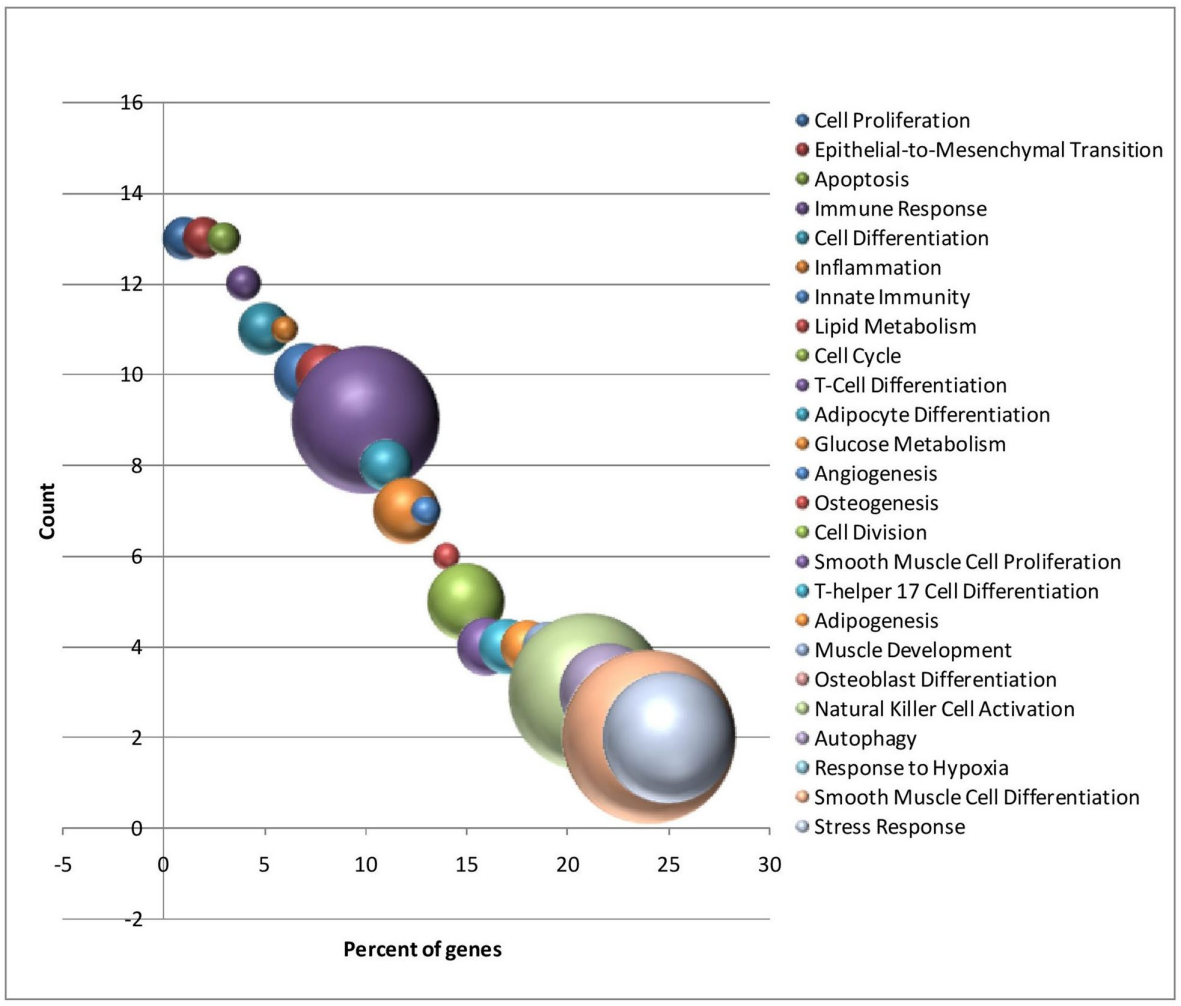
Top 25 functional categories for predicted target genes of differentially expressed miRNAs (log_2_ fold change ≥ ( ±) 3.0) using TAM 2.0^27^.

To underscore the most pertinent biological functions, all miRNAs with a fold change (FC) of ≥ ( ±) 3.0 were subjected to further analysis. A total of 45 miRNAs exhibited a log_2_ FC of ≥ ( ±) 3.0 (Table S3). These miRNAs belonged to the let-7, miRmiR-148 and miRmiR-33 families. The functional relevance of these miRNAs was determined using TAM 2.0^27^. The top 25 functional categories (p_adj_ ≤ 0.05, FDR ≤ 0.05) are depicted in Fig. 2. The enriched functional categories were cell proliferation, epithelial to mesenchymal transition, apoptosis, lipid metabolism, adipocyte differentiation, glucose metabolism, besides others.

### Network analysis

**Figure 3:**
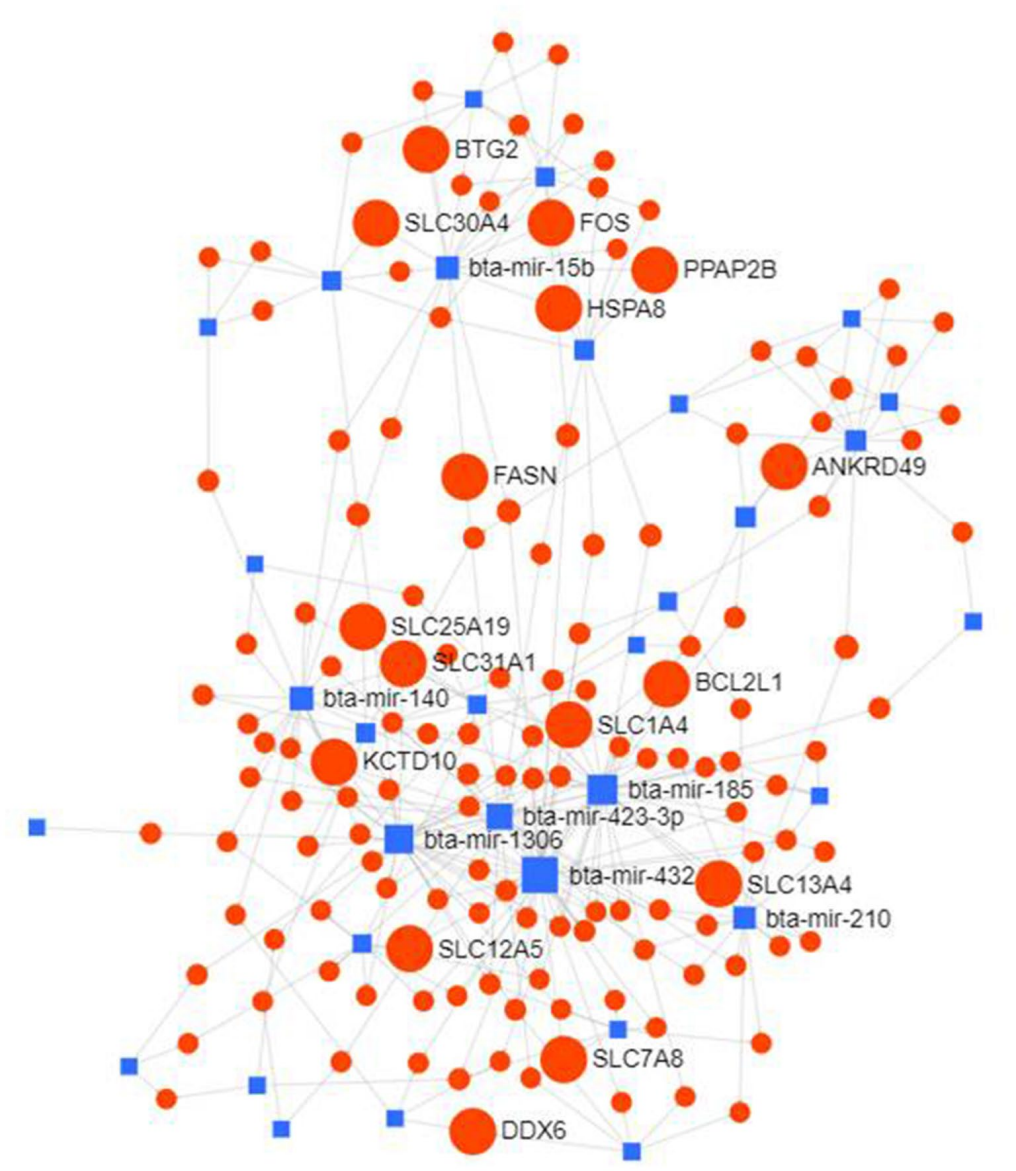
MicroRNA—target gene interaction network of differentially expressed miRNA (log_2_ FC ≥ ( ±) 3.0) constructed using miRNet.

**Figure 4:**
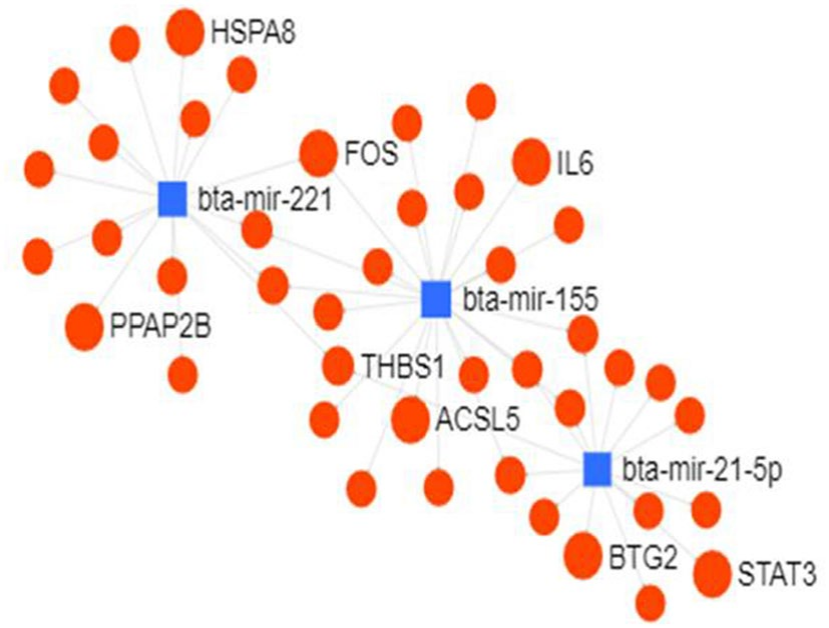
MicroRNA to target gene sub-network of differentially expressed miRNAs with more than 10 interactions created using miRNet.

**Figure 5:**
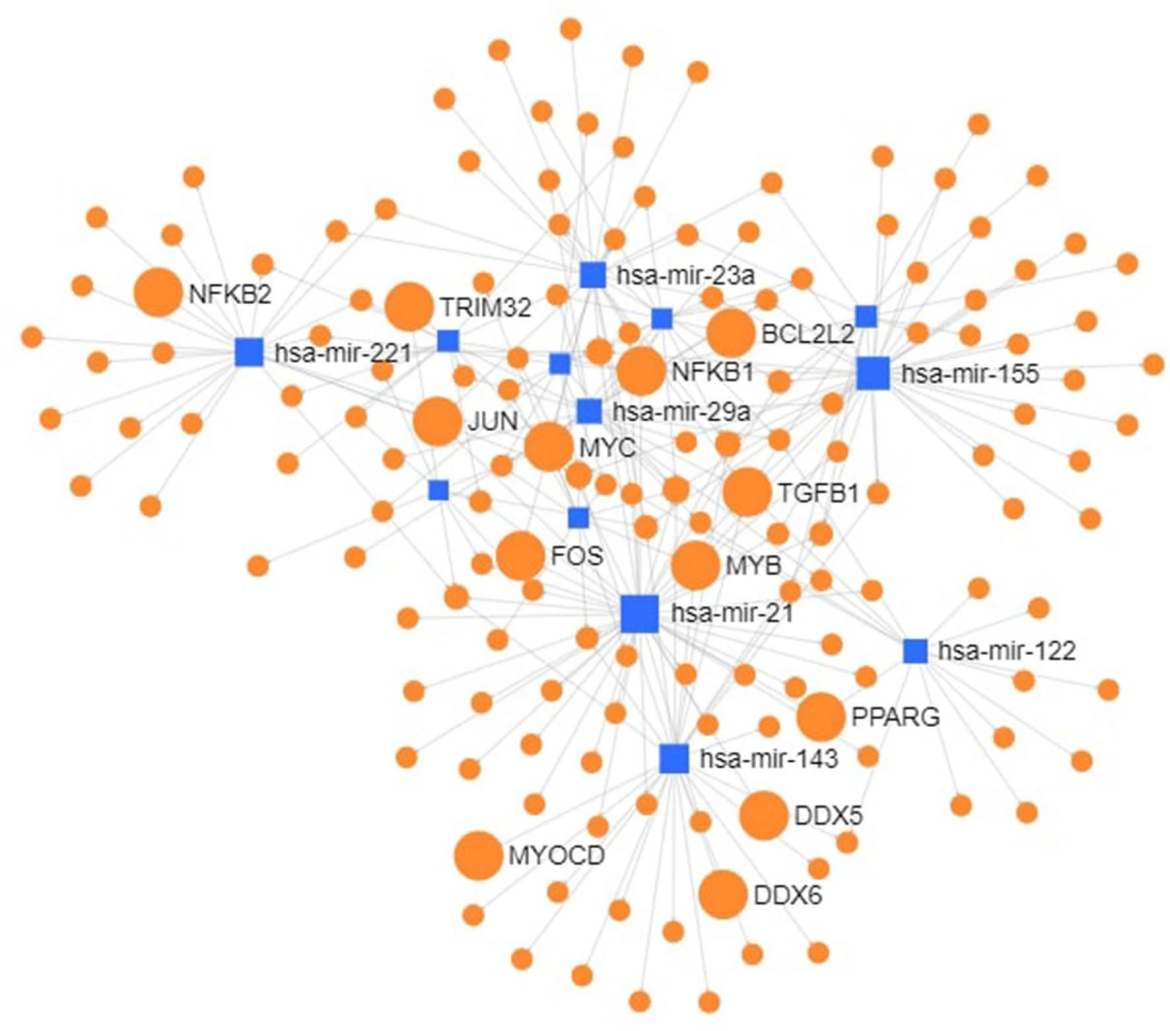
MicroRNA-transcription factors network of differentially expressed miRNAs with more than 10 interactions created using miRNet.

The differentially expressed miRNAs (≥ 3.0 log_2_ FC) were used for construction of interaction network for identification of important genes and transcription factors. The network consisted of 1044 nodes and each miRNA having more than 5 interactions (Fig. 3). Important genes identified in the network were *ANKRD49, BCL2L1, BTG2, DDX6, FASN, FOS, HSPA8, KCTD10, PPAP2B, SLC12A5, SLC1A4, SLC13A4* and *SLC25A19*. A sub-network was further visualized to ascertain the interactions between miRNAs with ≥ 10 degrees, having 46 nodes and 51 edges. (Fig. 4). MicroRNAs miR-21, miR-155, miR-143, miR-221, miR-23a, miR-29a, miR-122, miR-424, miR-29c, let-7b, miR-27b, miR-15a and let-7c were observed to be highly connected. The significant target genes thus detected included *HSPA8, FOS, ACSL5, BTG2* and *PPAP2B*. Another network based on transcription factors (human) as targets of these 13 highly connected miRNAs is shown in Fig. 5. Major transcription factors associated with these miRNAs were NFKB1, NFKB2, FOS, JUN, MYB, MYOCD, MYC, TGFB1, PPARG, BCL2L2, DDX5, DDX6.

### Validation by quantitative real time PCR (qRT-PCR)

**Figure 6:**
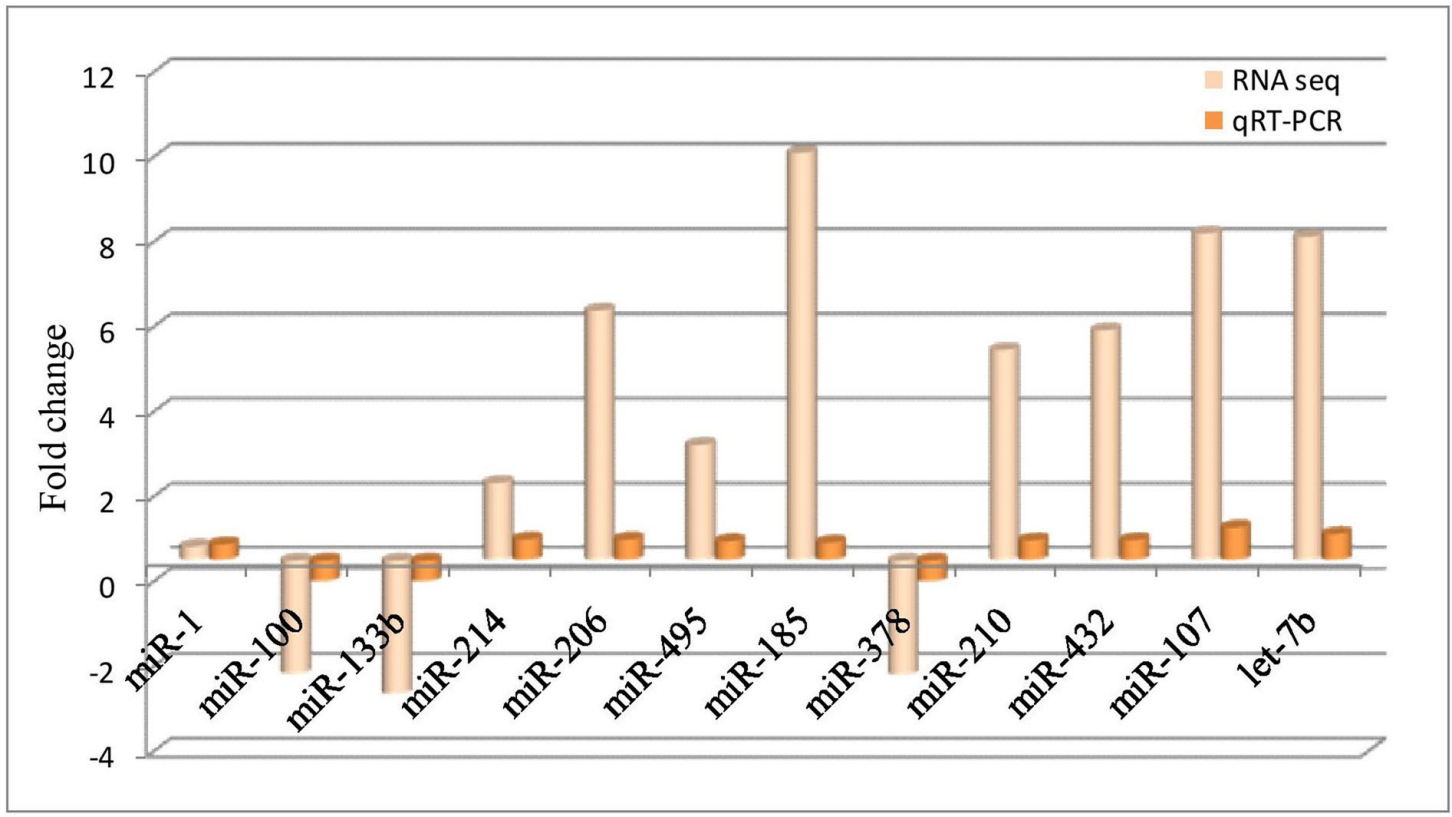
Validation of sequencing data by comparing log_2_ fold change between RNAseq and qRT-PCR data, for selected miRNAs across Bandur and local sheep. qRT-PCR data was normalized using *5S-rRNA* and *U6* genes**.**

Quantitative real time PCR was performed for validating the expression of some randomly selected differentially expressed miRNAs. The analysis revealed that the expression pattern (up-regulated or down-regulated) of miR-1, miR-100, miR-133b, miR-185, miR-206, miR-214, miR-378, miR-495-5p, miR-210, miR-432, miR-107 and let-7b was comparable by both RNA sequencing and qRT-PCR (Fig. 6).

## Discussion

Muscle fibres, intramuscular fat, nutrition, post mortem processing along with genetics play an important role in determining the quality of meat. Several genes have been reported to be associated with meat tenderness^28^, intramuscular fat^29^ and post mortem proteolysis^30^. The role of regulatory elements influencing the gene expression is gaining emphasis in recent times. MicroRNAs are one such class of regulatory molecules that post-transcriptionally affect the expression of genes. Knowledge of miRNAs in skeletal muscles of various livestock species provides the possibility of identifying key regulators of muscle traits that can be used as biomarkers^21,31^. Several studies have identified and profiled miRNAs in the skeletal muscles of sheep^11,12^. However, there is dearth of information on molecular regulators for meat type sheep in India. This study investigated the differential expression of miRNAs in Indian sheep with contrasting carcass and muscling traits^24^. MicroRNAs most abundantly expressed in Bandur and local sheep included miR-206, let-7b and miR-1 (> 50,000 RPKM). MiR-206 and miR-1 are important myomiRs known to target several genes influencing skeletal muscle development and differentiation^3^. Recent studies have expanded our understanding of the role of miR-1 and miR-206 in muscle development and disease^32^. Some of the genes regulated by miR-1are *HDAC4* and *YY1* that are directly involved in muscle development^33^. Moreover, miR-1 and miR-206 have been reported to be under transcription repression by *YY1*. They are involved in inhibition of expression of myostatin which leads to muscle hypertrophy in Texel sheep^12^. Both miR-1 and miR-206 were observed to be highly expressed in caprine skeletal muscles^9^. Although let-7 family of miRNAs is not specific to muscle tissue, it is highly conserved across species and mainly promotes differentiation during the developmental process^34,35^. In sheep, eight types of let-7 family genes have been reported^34^. Let-7b is known to mediate skeletal muscle growth in chicken^36^. Let-7 family was also observed to be highly expressed in lean pigs^37^. However, the exact role of let-7 family in muscle development is still not understood.

Among the up-regulated differentially expressed microRNAs, miR-185 along with miR-107 affects cell cycle regulation^38^. MiR-185 has also been implicated in regulating lipid metabolism and LDL uptake^39^. It has been reported that over expression of miR-107 inhibited bovine myoblasts differentiation and protected cells from apoptosis^40^. The down-regulated miR-486, in our study, is known to promote differentiation of myoblasts via PI3K/AKT signaling^41^ and has been reported to show higher expression during muscle differentiation^42^. However, it was also observed to be down-regulated in cattle^43^ and pig^7^. MiR-133a/b on the other hand promotes both proliferation and differentiation via MAPK1/MAPK3 signaling^44^. It also affects oxidative stress control and cell fate regulation^4^, while miR-10 is involved in protein metabolism^45^. Our results indicate that the differentially expressed miRNAs in Bandur and local sheep were mainly implicated in myogenesis and lipid metabolism. These observations are consistent with our previous study on mRNAs of the same animals^24^. Even though the shear force estimates between the two sheep populations under investigation were significantly different, we did not find miR-182, miR-183 and miR-338 to be differentially expressed. In contrast, these miRNAs were observed to be differentially expressed in cattle with contrasting estimated breeding values for shear force^21^. A comparison of the two studies however, would not be feasible as the study was specific to beef tenderness, while ours is a preliminary investigation into the differentially expressed miRNA in sheep populations with different carcass and muscling characteristics.

Of the total target genes identified, we focused only on those that are potentially related to muscling traits. As a consequence, the functional terms associated with these target genes were protein and lipid metabolism as well as muscle development. All the target genes (11062) were used for pathway analysis and the enriched pathways were also suggestive of protein and lipid metabolism. Other pathways associated with meat quality traits included PI3K-Akt signaling, EGFR, cellular response to stress, JAK STAT and focal adhesion. The major pathways relevant to skeletal muscles are Ras- Erk-MAPK, PI3K-Akt and calcineurium^46^. Current advances in our understanding of muscle development reveal that PI3K-Akt is a major signaling cascade for muscle differentiation and hypertrophy^47,48^. This signaling pathway has also been reported to stabilize the hypoxia inducible factor (HIF-1α), suggestive of its role in post-mortem protein proteolysis^49^.The PI3K-Akt pathway along with PPAR signaling was also enriched in bovine^50^ and porcine^51^ adipose tissue. It was identified as an enriched cluster of down-regulated genes in Bandur sheep^24^. EGFR is another widely investigated pathway that regulates myogenesis^52^. Besides, the PI3K-Akt signaling can be activated by the EGFR (a tyrosine kinase receptor) during cell proliferation and survival^53^. Although the JAK-STAT pathway regulates several hormones and cytokines, it is also known to enhance hypertrophy in the skeletal muscles^54^. Moreover, it leads to increased uptake of glucose, lipolysis and fatty acid oxidation by activating AMPK, PI3K and IL6 pathways^55^. The key pathways known to be involved in muscle metabolism and development were observed to be enriched in our study. The enrichment of cellular response to stress and fatty acid metabolism pathways corroborate our previous results on mRNA expression in Bandur sheep^24^.

If we consider only those target genes that are associated with meat tenderness, we observed that the genes *CAPN15, CASP3* and *HSPA8,* were targets of maximum number miRNAs. *HSPA8* and *CASP3* are involved in cellular stress response especially apoptosis^56^ while *CAPN15* is known for its role in muscle protein degradation^57^. Both caspase and calpain belong to a family of cysteine proteases that are intricately involved in apoptotic and necrotic pathways. Apoptosis or programmed cell death is a complex process mediated by the intrinsic (mitochondrial) and extrinsic (cell death) pathways. Caspases or cysteine proteases are major mediators of apoptosis. *CASP3* forms the link between the extrinsic and intrinsic pathways that brings about the morphological and biochemical changes in the cell in response to stress^58^. Although caspases have been extensively studied, the role calpain is still not well understood. However, calpains are important regulators of apoptosis having crosstalk with the caspase cascade^59^. *HSPA8* belongs to the family of heat shock proteins 70 (HSP70) that is induced in response to oxidative stress. Up-regulation of HSP70 hinders the activity of caspase thereby, inhibiting apoptosis^58^. Oxidative stress has been recognized as a major factor affecting post mortem proteolysis which in turn impacts muscle protein quality and shelf‐life of meat^60^. In light of these observations, it is reasonable to infer that post mortem proteolysis may play a crucial role in determining the muscle quality in the investigated sheep.

The network analysis revealed miR-21, miR-155, miR-143, miR-221 and miR-23a as the nodal miRNAs, with multiple targets. All of these miRNAs identified by the network analysis are implicated in development, proliferation and/or differentiation of skeletal muscles^61–63^. Although most of these associations are known in the context of human diseases mainly cancer, not enough evidence is available in support of their role in muscling traits. MiR-21, up-regulated in Bandur sheep (log_2_ FC = 4) formed the focal point of the network with 42 interactions. It has been revealed that miR-21 targets TGFβ1 via the PI3K-Akt -mTOR signaling in development of skeletal muscle of pig^64^. It has been acknowledged as an oncomiR that represses a number of genes of the apoptotic pathway^65^. It was also identified as one of the hub miRNA related to feed efficiency in Nellore cattle^43^. The PI3K signaling regulates not only growth and proliferation but also angiogenesis, cell survival and apoptosis. Transcription factors NFKB1, NFKB2, TGFB2^66^, MYC^67^ and BCL2L2^68^ have also been associated with apoptosis and cell survival in the PI3K signaling cascade. Post mortem proteolysis is mainly governed by the physiological processes of cell survival and apoptosis that contribute to tenderization of muscle protein^21,69^. Although the PI3K signaling is integrated into several cellular pathways, the identification of nodal miRNA and transcription factors in our study further lend support to its relevance in apoptosis and cell survival.

Molecular factors that repress the oxidative stress leading to apoptosis may find potential application in improvement of meat quality in addition to muscular disease. The hub miRNAs identified in our study form putative regulatory candidates for future research on meat quality traits in Indian sheep. Our results provide insight into the biological pathways and regulatory molecules in skeletal muscles of sheep.

## Materials and methods

### Samples

Four rams of Bandur and four local sheep, in the two-tooth stage (12–19 months) were selected. The phenotypes, carcass measurements and muscle tenderness of the animals used in this study have been previously described^24^. The animals were slaughtered according to standard commercial ‘*halal*’ procedures. All ethical norms and guidelines were followed, with approval from Institutional Animal Ethics Committee, ICAR-National Bureau of Animal Genetic Resources, Karnal, Haryana, India (F.No. NBAGR/IAEC/2017, dated 21.01.2017). Immediately after slaughter, the *longissimus thoracis* muscle was collected in RNALater solution.

### Micro RNA sequencing

Total RNA was extracted using TRIzol method and purified using RNeasy kit (Qiagen). Four biological replicates from Bandur as well as local sheep, with RIN value ≥ 7.0 (Agilent Bioanalyzer) were used for library preparation. Sequencing libraries were generated by Illumina Trueseq small RNA Sample Prep kit using 1 µg of total RNA. The libraries were normalized to a concentration of 2 nM using Tris–HCl 10 mM, pH 8.5. All the samples were purified, indexed, diluted and sequenced on Illumina HiSeq 2000 platform.

### Data analysis

FastQC (v 0.11.5)^70^ was used to assess the quality of the samples. Clean reads, after removal of adapters, were used to map the miRNA against the ovine genome assembly v4.0 (Oar_v4.0), available in NCBI), using miRNAkey^71^. miRDeep* software (v37.0.0) was used for identification of known and novel miRNAs in data^72^. Minimum free energy for novel miRNAs was calculated by using RNAfold tool (v2.4.3) from ViennaRNA package (v2.0)^73,74^. DESeq package (v1.28.0), which uses a negative binomial distribution, was used for differential analysis^75^. The gene expression differences between groups were tested using an exact test in this software. Expression levels of miRNA reads were normalized as reads per kilobase million (RPKM). Only those differentially expressed miRNA with p_adj_ ≤ 0.05 were used for further analysis.Target genes were identified using both human and cattle species in miRTarBase^76^. The functional and pathway categorization of the target genes was carried out using ConsensusPathDB^25,26^ and TAM 2.0^27^. The software miRNet^77,78^ was used for network construction.

### Validation by quantitative real time PCR (qRT-PCR)

Twelve random miRNAs from the list of differentially expressed miRNAs with p-value ≤ 0.05 were selected for qRT-PCR. Primer sequences were selected from literature or designed using sRNAPrimerDB software^79^ (Table S4). Stem loop PCR method was used for amplification^80^. Total RNA including miRNA was extracted from the skeletal muscle tissue of Bandur and Local sheep using TRIzol reagent as per the manufacturer’s instructions. The samples were purified and enriched using miRNeasy Micro Kit (Qiagen) to remove genomic DNA contamination. cDNA was synthesized from 250 ng purified RNA isolated from *longissimus thoracis* of local and Bandur sheep using Thermo Scientific RevertAid First Strand cDNA Synthesis Kit, as per manufacturer’s protocol. Gene amplification by qRT-PCR was performed in Roche RT system (2 step PCR). A 10 μl reaction mixture consisting of 25 ng of template, 0.3 μl of forward and reverse primers each (2 nmol/μl) and 5 μl of SYBR green mix (2x) was prepared. The amplification programme was 95 °C for 10 min, followed by 45 cycles of 95 °C for 10 s, 58 °C for 10 s and 72 °C for 10 s. A standard curve calculation by using four points of cDNA serial dilutions was used to determine the efficiency of reaction. All reactions were performed in triplicates. 5S-*rRNA* and *U6 snRNA* were used as internal control genes.

Normalization for all the miRNA was done using the geometric mean of both the reference genes and 2^−ΔΔCt^ method was used for calculating the relative gene expression^81^.

## Supplementary information

Supplementary file1
